# Clinical features and risk factors for interstitial lung disease spreading in low-dose irradiated areas after definitive radiotherapy with or without durvalumab consolidation therapy for patients with non-small cell lung cancer

**DOI:** 10.1186/s13014-023-02276-7

**Published:** 2023-05-22

**Authors:** Mai Sakagami, Haruo Inokuchi, Nobutaka Mukumoto, Hiroshige Itoyama, Nobunari Hamaura, Mutsumi Yamagishi, Naoki Mukumoto, Shogo Matsuda, Daijiro Kabata, Keiko Shibuya

**Affiliations:** 1 Department of Radiation Oncology, Graduate School of Medicine, Osaka City University, Osaka, Japan; 2 Department of Radiation Oncology, Graduate School of Medicine, Osaka Metropolitan University, Osaka, Japan; 3 Department of Medical Statistics, Graduate School of Medicine, Osaka Metropolitan University, Osaka, Japan

**Keywords:** Non-small cell lung cancer, Radiotherapy, Chemoradiotherapy, Durvalumab, Radiation pneumonitis, Interstitial lung disease, Risk factor, Diabetes

## Abstract

**Background:**

The current standard of care for patients with unresectable locally advanced non-small cell lung cancer (NSCLC) is chemoradiotherapy (CRT) combined with durvalumab consolidation therapy. However, radiotherapy (RT) always carries the risk of radiation pneumonitis (RP), which can preclude durvalumab continuation. In particular, the spread of interstitial lung disease (ILD) in low-dose areas or extending beyond the RT field often makes it difficult to determine the safety of continuation or rechallenging of durvalumab. Thus, we retrospectively analyzed ILD/RP after definitive RT with and without durvalumab, with assessment of radiologic features and dose distribution in RT.

**Methods:**

We retrospectively evaluated the clinical records, CT imaging, and radiotherapy planning data of 74 patients with NSCLC who underwent definitive RT at our institution between July 2016 and July 2020. We assessed the risk factors for recurrence within one year and occurrence of ILD/RP.

**Results:**

Kaplan-Meier method showed that ≥ 7 cycles of durvalumab significantly improved 1-year progression free survival (PFS) (p < 0.001). Nineteen patients (26%) were diagnosed with ≥ Grade 2 and 7 (9.5%) with ≥ Grade 3 ILD/RP after completing RT. There was no significant correlation between durvalumab administration and ≥ Grade 2 ILD/RP. Twelve patients (16%) developed ILD/RP that spread outside the high-dose (> 40 Gy) area, of whom 8 (67%) had ≥ Grade 2 and 3 (25%) had Grade 3 symptoms. In unadjusted and multivariate Cox proportional-hazards models adjusted for V_20_ (proportion of the lung volume receiving ≥ 20 Gy), high HbA1c level was significantly correlated with ILD/RP pattern spreading outside the high-dose area (hazard ratio, 1.842; 95% confidence interval, 1.35–2.51).

**Conclusions:**

Durvalumab improved 1-year PFS without increasing the risk of ILD/RP. Diabetic factors were associated with ILD/RP distribution pattern spreading in the lower dose area or outside RT fields, with a high rate of symptoms. Further study of the clinical background of patients including diabetes is needed to safely increase the number of durvalumab doses after CRT.

## Introduction

Concurrent chemoradiotherapy (CRT) or radiotherapy (RT) has been recommended for patients with unresectable locally advanced non-small cell lung cancer (NSCLC). The PACIFIC study has recently shown good prognostic prolongation effect of the combination of CRT and durvalumab consolidation therapy [1–3], which has become a standard treatment for these patients. However, RT always carries the risk of radiation pneumonitis (RP) and is sometimes fatal [4]. Programmed cell death-ligand 1 (PD-L1) inhibitor can also cause interstitial lung disease (ILD) [5]. Although the rate of severe pneumonitis was similar in both arms in the PACIFIC trial, it should be noted that for several reasons certain patients were excluded after CRT in this trial. In clinical practice, it has been reported that 23% of patients who were eligible for initiation of CRT did not meet the criteria for the PACIFIC study after CRT, and that RT with V20 (volume of lung parenchyma that receives 20 Gy or more) greater than 35% was associated with ineligibility [6]. Therefore, the safety and risk factors of RP after definitive CRT with durvalumab consolidation therapy have not been sufficiently confirmed in the real world.

In contrast, it may be possible to rechallenge durvalumab when obvious RP is present, even Grade 2 or higher, and in fact a previous report has suggested its safety [7]. However, ILD/RP that occurs in the low-dose irradiated area or that which has spread beyond the irradiation field tends to become more serious, and caution is required for rechallenging. In addition, it is often difficult to distinguish between drug-induced and radiation-induced ILD in such patients, which makes it more difficult to determine whether durvalumab can be rechallenged. As intensity-modulated radiation therapy (IMRT), which tends to increase the lung volume exposed to lower doses, becomes widely used for the treatment of lung cancer, it is expected that it will be increasingly difficult to determine the cause of ILD occurring in low-dose irradiated regions.

Thus, we retrospectively analyzed the incidence, severity, and risk factors for ILD/RP after definitive RT with and without durvalumab, in parallel with an assessment of the radiologic distribution pattern and changes of interstitial shadows over time of ILD/RP in relation to dose distribution in RT by computed tomography (CT). We also evaluated early outcomes of RT/CRT in patients with NSCLC before and after approval of durvalumab.

## Methods

### Patients, data collection, and clinical endpoints

We retrospectively reviewed the clinical records, radiographic information, and radiotherapy treatment planning data of patients who received curative-intent radiotherapy for histologically confirmed NSCLC at our institution between 1 July 2016 and 31 July 2020. Data collected included patient age, sex, histology, clinical stage of lung cancer (UICC version 8), Brinkman index, concurrent chemotherapy (with/without), durvalumab (with/without), total lung volume, pulmonary fibrosis score [8], pulmonary emphysema score [9], Krebs von den Lungen-6 (KL-6), lactate dehydrogenase (LDH), C-reactive protein (CRP), and hemoglobin A1c (HbA1c, an indicator of glycemic control) level before treatment. Radiotherapy planning data included the dose–volume metrics of lung: V_5_, V_20_, Vs5, and mean lung dose (MLD). Vx signified the proportion of the volume receiving ≥ x Gy. Vs5 was defined as the absolute lung volume spared from a 5 Gy dose. Lung volume in the dose–volume metrics was defined as the total lung volume minus the GTV. In patients for whom boost irradiation was planned on the re-imaged CT data set, dose–volume histogram analysis was performed using total dose as the sum of the initial and boost plans reproduced on the initial CT set.

Regardless of the association with RT, all interstitial shadows observed after definitive RT were extracted as ILD. ILD grading was based on pneumonitis grade according to the National Cancer Institute Common Terminology Criteria for Adverse Events (NCI-CTCAE), version 5.0. The number of days until the onset of ILD was calculated from the date of completion of radiotherapy. For asymptomatic Grade 1 ILD, date of onset was defined as the date at which the lung volume showing interstitial shadows had become the largest. The date of ≥ Grade 2 ILD was defined as the date of imaging at which the respiratory physician recognized ILD as requiring therapeutic intervention with steroids based on both the symptoms and the imaging findings.

The CT images that showed findings of ILD were fused with the dose distribution images and evaluated by two radiation oncologists. The distribution pattern of interstitial shadow was then classified either as type I: no obvious shadow or shadows distributed only within the high-dose (> 40 Gy) irradiated zone, or type II: shadows distributed outside the high-dose (> 40 Gy) irradiated zone.

The primary endpoint was assessment of risk factors and characteristics of symptomatic ILD/RP in patients treated with RT/CRT with or without durvalumab. The secondary endpoint was the early control rate in these patients.

This study was approved by our institutional review board. Informed consent was obtained by the opt-out method in accordance with the disclosure document.

### Statistical analysis

ILD were classified into two groups according to severity of disease: Grade 0–1, and Grade 2 or higher (symptomatic), and according to distribution of interstitial shadow: type I and type II. We summarized the distributions of the candidate factors affecting the severity and distribution of ILD using medians and counts within each ILD groups for continuous variables and categorical variables, respectively among 74 patients. The distributions of the numerical factors were compared using Mann-Whitney U-test whereas the proportion of the categorical factors compared using Chi-square test or Fisher-exact test.

Furthermore, to assess the association between the candidate factors and ILD type considering the ILD onset timing, we performed unadjusted Cox proportional-hazard regression analyses for all candidate factors. In addition, we conducted the Cox proportional-hazard regression analyses with adjustment for V_20_, because several previous studies have shown the association between V_20_ and the severe radiation pneumonitis [10–12]. For avoiding multicollinearity with V_20_, the adjustments were not performed for V_5_ and MLD. For seven non-diabetic patients whose HbA1c was not measured at the start or end of RT, Cox proportional-hazard regression analyses were performed by substituting 5.8%, the mean value of HbA1c in non-diabetic patients. For eight patients with Grade 0 ILD, Cox proportional-hazard regression analyses were performed by substituting 220 days for ILD onset timing.

Finally, we examined the association between the candidate factors and the recurrence within 1 year using Mann-Whitney U-test, Chi-square test or Fisher-exact test. Moreover, to evaluate the impact of durvalumab on the recurrence within 1 year, we estimated PFS rate using Kaplan–Meier method, and then, log-rank test were conducted for comparison of the PFS rate between the patient groups divided into the following three groups: those who received durvalumab for a long time (> 3 cycles), those who could not receive durvalumab or who received three cycles or fewer, and those who did not receive durvalumab because it was not yet approved. Patients with no recurrence were censored at 400 days.

All hypothesis tests were conducted based on two-sided 5% significance level with the SPSS package, version 27.0 (IBM Corp, Chicago, Illinois, United States of America).

## Results

### Patient and treatment characteristics

A total of 74 patients who were treated with RT of more than 50 Gy with curative intent were included in this study. Eighteen patients (24%) who did not receive chemotherapy due to renal function, age, other comorbidities, or patient unwillingness were also included. No patients received neoadjuvant or adjuvant chemotherapy. Most patients received concurrent carboplatin/paclitaxel (43%) or cisplatin/vinorelbine (27%). After completing concurrent chemoradiotherapy, 23 (31%) patients proceeded to consolidation durvalumab therapy (10 mg/kg, every 2 weeks, up to 1 year). All patients were treated with three-dimensional conformal radiotherapy (3D-CRT) and 3 (4%) were treated with IMRT limited to the boost dose fields. In 68 (82%) patients, the radiation treatment plan was changed to a boost plan after approximately 40 Gy of irradiation. Table 1 lists the patient, tumor, and treatment characteristics.

**Table 1:** Patient characteristics

Characteristic	No. (%) Or Median (range)
**Age (years)**	69 (63–76)
**Sex**	
Male	51 (69%)
Female	23 (31%)
**Smoking history**	
Current or past smoker	66 (89%)
Never smoker	8 (11%)
**Brinkman index**	840 (500–1105)
**LDH (U/L)**	207 (177–235)
**CRP (mg/dL)**	0.35 (0.08–1.43)
**HbA1c (%)**	5.9 (5.6–6.5)
**Glucose (mg/dL)**	105 (95–121)
**KL-6 (U/mL)**	315 (219–586)
**Histology**	
Adenocarcinoma	37 (50%)
Squamous cell carcinoma	26 (35%)
NSCLC, NOS	11 (15%)
**Clinical stage**	
I	2 (3%)
II	5 (7%)
IIIA	22 (30%)
IIIB	25 (34%)
IIIC	13 (18%)
IVA	3 (4%)
Recurrent	4 (5%)
**Chemotherapy**	
Carboplatin/paclitaxel	32 (43%)
Cisplatin/vinorelbine	20 (27%)
Other	4 (5%)
None	18 (24%)
**Durvalumab (+)**	23 (31%)
**RT dose**	
50 Gy (2.5 Gy/1 fr)	1 (1%)
60 Gy	54 (73%)
66 Gy	19 (26%)
**IMRT**	3 (4%)
**V**_**20**_ **(%)**	22.3 (17.7–30.5)
**V**_**5**_ **(%)**	37.6 (29.0–47.0)
**MLD (Gy)**	12.6 (10.2–16.4)
**Vs5 (cc)**	2089 (1476–2626)
**Lung volume (cc)**	3371 (2927–4247)
**ILD onset timing (days)**	85 (59–120)

Abbreviations

LDH: Lactate dehydrogenase

CRP: C-reactive protein

HbA1c: hemoglobin A1c

KL-6: Krebs von den Lungen-6

NSCLC, NOS: non-small cell lung cancer, not otherwise specified

RT: radiotherapy

IMRT: intensity-modulated radiation therapy

Vx: proportion of the volume receiving ≥ x Gy

MLD: mean lung dose

Vs5: absolute lung volume spared from a 5 Gy dose

ILD: interstitial lung disease

### ILD

Two patients diagnosed with drug-induced pneumonitis (Grade 2 and Grade 3 in one patient each) and treated accordingly were included in the analysis as ILD. Sixty-six patients (89%) were diagnosed with ≥ Grade 1 ILD, 19 (25.7%) with symptomatic ≥ Grade 2, and 7 (9.5%) with ≥ Grade 3. The comparison of the patient, tumor, and treatment characteristics between patients with Grade 0–1 (n = 55) and those with ≥ Grade 2 ILD (n = 19) are shown in Table 2. Among patients with stage III NSCLC treated with CRT, 6 (26%) patients who were treated with durvalumab and 4 (19%) patients who were not treated with durvalumab (because it was before approval) were diagnosed with ≥ Grade 2 ILD.

**Table 2:** Clinical factors for severity and distribution pattern of interstitial lung disease

		Grade 0–1 ILD	≧Grade 2 ILD	p value		Type I	Type II	p value
		(n = 55)	(n = 19)			(n = 62)	(n = 12)	
**Age (years)**		68	71	0.622		68.5	71	0.597
**Sex, n**	**Male**	36	15			41	10	
	**Female**	19	4	0.273		21	2	0.204
**Brinkman index**		820	990	0.637		783	1000	0.27
**Pulmonary fibrosis score, n**	**0–1**	54	15			58	11	
	**≥ 2**	1	4	0.014		4	1	0.598
**Pulmonary emphysema score**		1	0	0.894		1	0	0.794
**LDH (U/L)**		197	233	0.015		198	226	0.016
**CRP (mg/dL)**		0.23	1.05	0.017		0.245	1.05	0.105
**HbA1c (%)**		5.9	6.1	0.29		5.85	6.9	0.002
**Glucose (mg/dL)**		102	106	0.26		102	131.5	0.02
**KL-6 (U/mL)**		324	313	0.821		295	439	0.312
**Clinical stage of lung cancer**		IIIB	IIIB	0.599		IIIB	IIIB	0.654
**Chemotherapy, n**	**–**	16	2			15	3	
	**+**	39	17	0.09		47	9	0.604
**Durvalumab, n**	**–**	38	13			44	7	
	**+**	17	6	0.957		18	5	0.293
**V**_**20**_ **(%)**		20.8	30.2	0.002		21.2	28.2	0.086
**V**_**5**_ **(%)**		35.6	46.5	0.01		35.9	41.4	0.356
**MLD (Gy)**		11.6	15.2	0.005		12.1	15	0.21
**Vs5 (cc)**		2224	1560	0.083		2089	2052	0.912
**Lung volume (cc)**		3389	3238	0.376		3371	3418	0.907
**ILD onset timing (days)**		93	61	0.007		93	39	0.001

Abbreviations

LDH: Lactate dehydrogenase

CRP: C-reactive protein

HbA1c: hemoglobin A1c

KL-6: Krebs von den Lungen-6

Vx: proportion of the volume receiving ≥ x Gy

MLD: mean lung dose

Vs5: absolute lung volume spared from a 5 Gy dose

ILD: interstitial lung disease

Type I: shadows distributed only within the high-dose (> 40 Gy) irradiated area

Type II: shadows distributed outside the high-dose (> 40 Gy) irradiated area

**Fig. 1:**
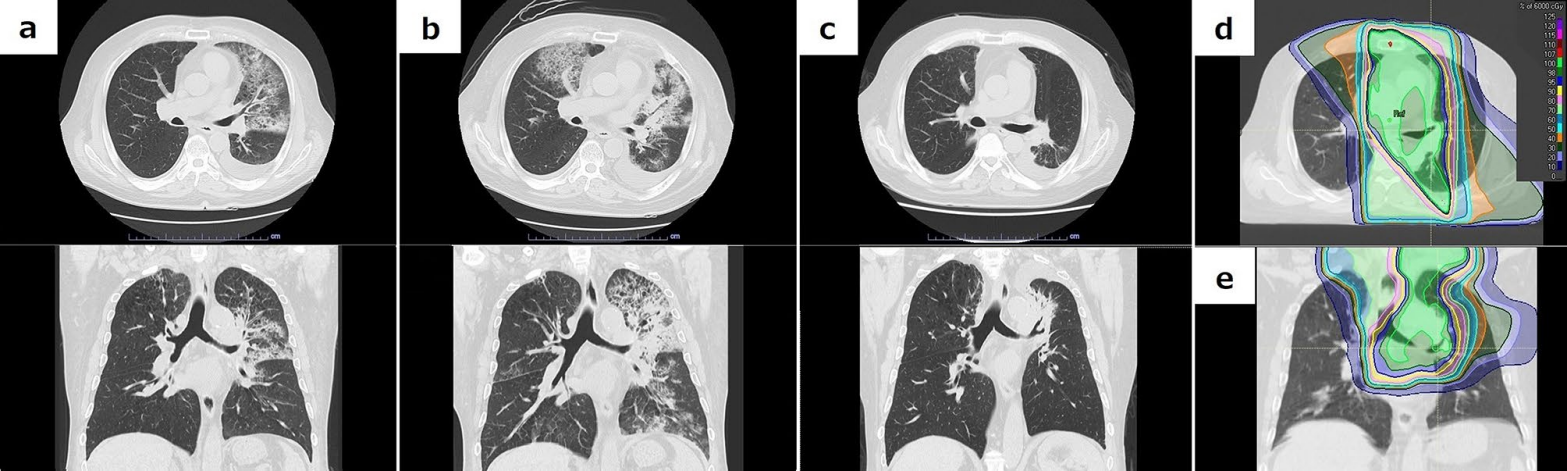
Axial (upper) and coronal (lower) computed tomography (CT) images of a representative patient Interstitial lung disease/radiation pneumonitis (ILD/RP) arising from the low-dose irradiated area after chemoradiotherapy (CRT) in patient #10 in Table 3. **(a):** On the 37th day after the completion of CRT, the patient had received 3 cycles of durvalumab before discontinuing due to diagnosis of Grade 2 ILD/RP. The shadow disappeared soon after initiation of prednisone (PSL, 80 mg/day = 1 mg/kg/day). **(b):** On the 89th day, when PSL was being tapered to 20 mg/day, infiltrative shadows and ground-glass opacities reappeared and spread over the irradiated area with worsened dyspnea, leading to diagnosis of Grade 3 ILD. **(c):** On the 173rd day, the infiltrative shadow and ground-glass opacities disappeared after increasing the dose of PSL, but fibrosis appeared limited to the high-dose irradiated areas. **(d):** axial and **(e):** coronal CT images show the radiotherapy dose distribution in this patient

Recurrent ILD occurred after durvalumab administration in one patient, in whom interstitial shadows were seen inside and outside the irradiated field throughout the course of symptomatic lung disease. Analysis of the radiographic changes and dose distribution in RT in all patients revealed type I pattern in 62 (84%) patients and type II pattern in 12 (16%) patients. Of the 12 patients with type II, 8 (67%) had ≥ Grade 2 ILD, including 3 (25%) who had Grade 3 symptoms. The incidence of ≥ Grade 2 ILD was significantly higher in type II pattern than in type I pattern (p = 0.001). Looking further at the distribution of infiltrating shadows on the respective CT images, these shadows were also seen even in irradiated fields less than 10 Gy in 4 patients, and three of them had similar shadows outside the irradiated area. Of the 4 patients, 1 had Grade 2 and 2 had Grade 3 symptoms in ILD; in all 4 patients, however, intense consolidation was observed only in the high-dose irradiated area during the subsequent course of treatment. Figure 1 shows representative CT images and dose distribution in these patients.

**Table 3:** Characteristics of patients with ILD distributed outside the high-dose (> 40 Gy) irradiated area (type II)

Patient characteristic				Treatment					ILD	
patient		HbA1c (%)		chemotherapy	durvalumab	V_20_ (%)	V_5_ (%)		ILD onset timing^1)^ (days)	NCI-CTCAE version 5.0 Grade
1		8.2		–	–	30.4	46.5		126	3
2		9.1		–	–	10.8	17.7		28	1
3		8.1		+	–	40.6	55.8		28	3
4				+	+	39.3	70.3		73	2
5		6.5		–	–	27.0	39.7		11	1
6		6.9		+	–	21.9	34.0		41	2
7		7.8		+	+	28.0	40.7		33	1
8		5.5		+	+	24.2	36.8		50	2
9		6.2		+	–	31.4	42.1		9	2
10		11.5		+	+	28.4	46.7		37	2
11		5.7		+	+	15.6	26.4		122	3
12		6.2		+	–	32.0	46.2		63	1

Abbreviations

ILD: interstitial lung disease

CT: computed tomography

NCI-CTCAE: National Cancer Institute Common Terminology Criteria for Adverse Events

HbA1c: hemoglobin A1c

Vx: proportion of the volume receiving ≥ x Gy

1) ILD onset timing: days from RT to the onset and/or diagnosis of ILD

In patients with type II distribution, median lung V_20_ was 28.2% and median lung V_5_ was 41.4%. In comparison of patient characteristics and treatment factors between type I and type II patient groups (Table 2), Mann-Whitney U-test showed no significant difference in terms of dose–volume parameters, but significant difference was seen in the timing of onset of ILD. Furthermore, no significant differences were seen in the administration of chemotherapy and durvalumab when using unadjusted Cox proportional-hazards models and V20 as a covariate. However, significant differences were shown in the levels of LDH, HbA1c, and glucose level (Table 4). LDH was also a significant factor in ≥ Grade 2 ILD. These results suggest that HbA1c and glucose level, which are indicators of diabetes, might be useful predictors of ILD extending beyond the high-dose irradiated field, independently of the dose–volume parameter.

**Table 4:** Unadjusted and V_20_ adjusted Cox proportional-hazards analyses of clinical factors of type II ILD

	Unadjusted				V_20_ adjusted		
	HR	95%CI	p value		HR	95%CI	p value
**Age (years)**	1.012	0.96–1.07	0.672		1.004	0.95–1.06	0.894
**Sex**	0.371	0.08–1.70	0.201		0.404	0.09–1.85	0.243
**Brinkman index**	1.000	1.000-1.001	0.297		1.000	1.000-1.001	0.437
**Pulmonary fibrosis score**	0.512	0.07–4.05	0.525		1.024	0.12–8.94	0.983
**Pulmonary emphysema score**	1.014	0.61–1.69	0.957		1.038	0.63–1.72	0.885
**LDH (U/L)**	1.010	1.005–1.015	< 0.001		1.009	1.004–1.014	< 0.001
**CRP (mg/dL)**	1.146	0.98–1.34	0.091		1.133	0.97–1.33	0.126
**HbA1c (%)**	1.896	1.38–2.60	< 0.001		1.842	1.35–2.51	< 0.001
**Glucose (mg/dL)**	1.017	1.004–1.031	0.011		1.017	1.003–1.031	0.018
**KL-6 (U/mL)**	1.000	0.998–1.002	0.962		1.001	0.998–1.003	0.666
**Clinical stage of lung cancer**	1.104	0.71–1.71	0.659		1.131	0.74–1.73	0.571
**Chemotherapy**	1.185	0.32–4.38	0.799		1.377	0.37–5.16	0.635
**Durvalumab**	0.690	0.22–2.18	0.527		0.576	0.18–1.85	0.354
**V**_**20**_**(%)**l1195	1.070	0.998–1.148	0.058				
**V**_**5**_ **(%)**	1.021	0.98–1.06	0.323				
**MLD (Gy)**	1.120	0.97–1.29	0.120				
**Vs5 (cc)**	1.000	0.999–1.001	0.745		1.001	1.000-1.002	0.082
**Lung volume (cc)**	1.000	0.999–1.001	0.855		1.000	1.000-1.001	0.292

Abbreviations

Type II: shadows distributed outside the high-dose (> 40 Gy) irradiated area

HR: hazard ratio

ILD: interstitial lung disease

LDH: Lactate dehydrogenase

CRP: C-reactive protein

HbA1c: hemoglobin A1c

KL-6: Krebs von den Lungen-6

Vx: proportion of the volume receiving ≥ x Gy

MLD: mean lung dose

Vs5: absolute lung volume spared from a 5 Gy dose

### Survival

**Fig. 2:**
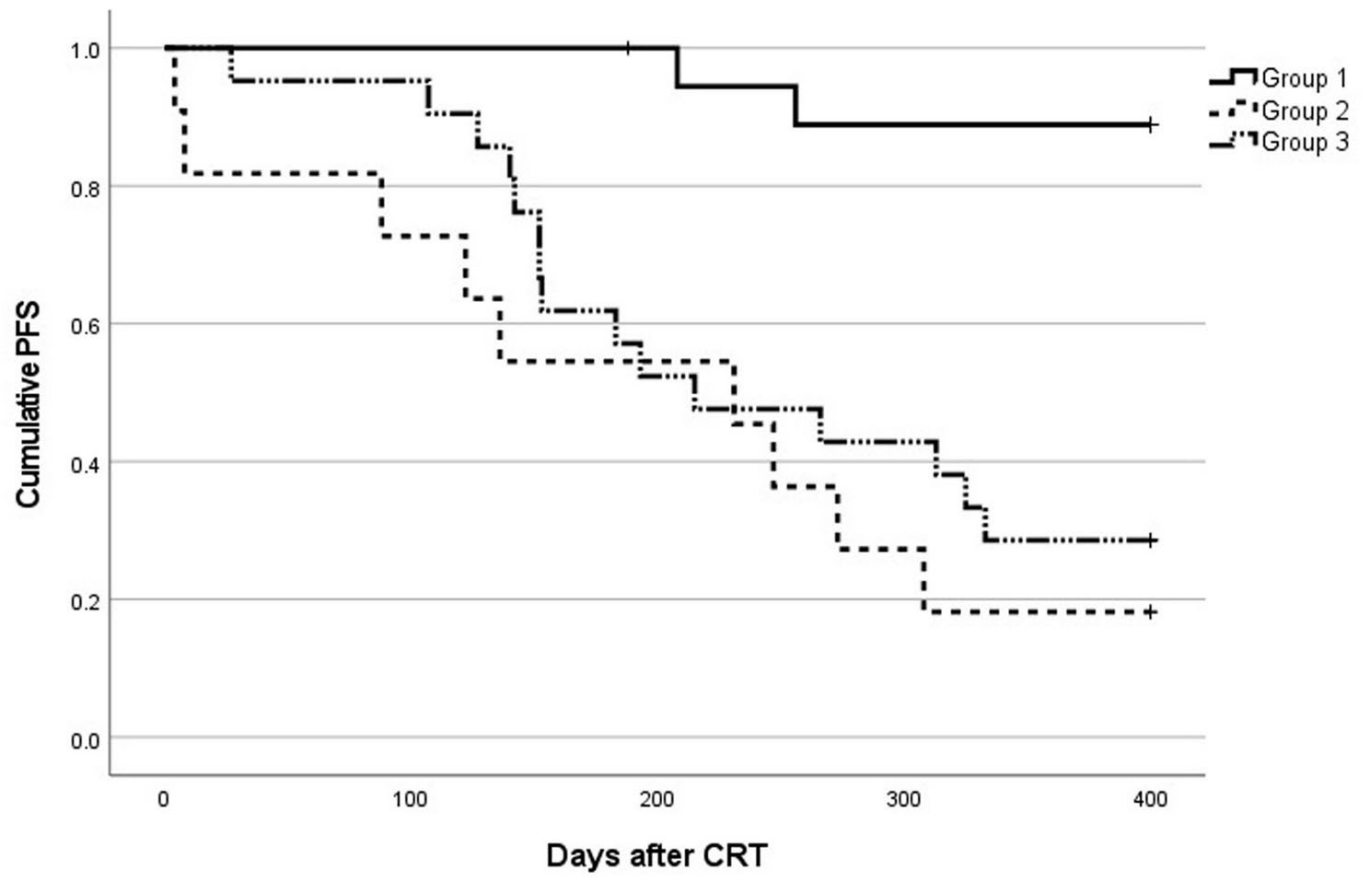
Kaplan–Meier curves for progression-free survival Group 1: 19 patients who received adequate durvalumab dosing Group 2: 7 patients who could not receive durvalumab after its approval, and 4 patients who received ≤ 3 cycles of durvalumab Group 3: 21 patients who did not receive durvalumab because it had not yet been approved PFS, progression-free survival; CRT, chemoradiotherapy

Clinical stage of NSCLC, and administration of durvalumab were significant factors in the recurrence within 1 year in univariate analysis (Table 5). To analyze the association between the number of durvalumab doses and 1-year progression-free survival (PFS), we extracted 51 patients with stage III NSCLC and divided them into three groups. Group 1 consisted of 19 patients who received durvalumab for more than three cycles (minimum number of cycles was 7). Group 2 included 7 patients who could not receive durvalumab after its approval (Of 7 patients, 5 did not receive it because they developed RP), and 4 patients who received ≤ 3 cycles. Group 3 included 21 patients who did not receive durvalumab because it had not yet been approved. One-year PFS was 90.0%, 18.2%, and 28.6%, respectively, in these three groups (Fig. 2). The log-rank test detected significant differences in PFS between Group 1 and Group 2, and between Group 1 and Group 3 (p < 0.001). No significant difference was detected between Group 2 and Group 3 (p = 0.346).

**Table 5:** Clinical factors for recurrence within 1 year

		No recurrence	Recurrence	p value
		(n = 39)	(n = 35)	
**Age (years)**		67	70	0.105
**Sex, n**	Male	29	22	
	Female	10	13	0.286
**Brinkman index**		800	870	0.795
**Pulmonary fibrosis score, n**	0–1	37	32	
	≥ 2	2	3	0.448
**Pulmonary emphysema score**		0	1	0.255
**LDH (U/L)**		192	214	0.086
**CRP (mg/dL)**		0.22	0.54	0.089
**HbA1c (%)**		5.9	5.8	0.484
**Glucose (mg/dL)**		103	105	0.803
**KL-6 (U/mL)**		324	288	0.545
**Clinical stage of lung cancer**		IIIB	IIIB	0.029
**Chemotherapy, n**	–	7	8	
	+	32	27	0.814
**Durvalumab, n**	–	21	30	
	+	18	5	0.003
**V**_**20**_ **(%)**		20.3	24.1	0.378
**V**_**5**_ **(%)**		34.6	39.0	0.458
**MLD (Gy)**		11.8	13.0	0.414
**Vs5 (cc)**		2183	1905	0.570
**Lung volume (cc)**		3389	3369	0.721
**ILD onset timing (days)**		90.5	85.0	0.582

Abbreviations

LDH: lactate dehydrogenase

CRP: C-reactive protein

HbA1c: hemoglobin A1c

KL-6: Krebs von den Lungen-6

Vx: proportion of the volume receiving ≥ xGy

MLD: mean lung dose

Vs5: absolute lung volume spared from a 5 Gy dose

ILD: interstitial lung disease

## Discussion

The results of the present analyses did not rule out the availability of durvalumab after chemoradiotherapy as an important factor in survival, as has been reported in several previous studies.

Initially, there was concern that durvalumab might increase the frequency of severe pneumonitis, but the results of the PACIFIC trial showed that the frequency of Grade 3 or higher pneumonitis was 4.7% in the durvalumab group and 5.1% in the placebo group, with no significant difference [13]. In contrast, RP after CRT is the main adverse event that can affect the availability of durvalumab administration. A retrospective Japanese multicenter observational study showed that the timing of incidence of pneumonitis was 10–12 weeks after completing CRT, which coincided with the period of durvalumab maintenance therapy [14]. Therefore, every effort should be made to reduce ≥ Grade 2 radiation pneumonitis as much as possible, and it is important to understand the pathophysiology and identify risk factors of pneumonitis after treatment.

Furthermore, when interstitial shadows are present in the lower-dose region of RT during durvalumab consolidation, it is often difficult to distinguish RP from drug-induced pneumonitis. As such cases tend to be severe, even as the more common RP, it is often difficult to determine whether durvalumab can be maintained after the inflammation has resolved. To explore the pathogenesis of such cases and ultimately prevent the occurrence of severe ILD/RP, we examined patient background factors, treatment-related parameters including RT, and the imaging features of dose distributions and changes of lung shadows over time, between patients with and without ILD/RP.

Of all patients, 89% showed the appearance of some interstitial shadow in the irradiated field after RT, and 26% had ≥ Grade 2 ILD/RP. In univariate analysis, the dose parameters (V_20_, V_5_, MLD) and timing of onset of pneumonitis were significant factors in the occurrence of ≥ Grade 2 ILD/RP, in agreement with several previous studies [4, 10–12, 15–18]. In the present study, durvalumab itself was not detected as a significant factor for increasing ILD, whereas the results of the imaging study on dose and ILD distribution found 12 patients (16%) with interstitial shadows in the lower-dose area, in whom the rate of symptomatic pneumonitis was 67%. Four of these patients (33%) also showed interstitial shadows in the lower-dose region below 10 Gy, including three patients who had similar shadows outside the irradiated area. In these four patients, drug-induced pneumonitis was also a differential at that time, but all subsequently showed strong fibrosis only in the high-dose area, suggesting that radiation was at least one of the factors affecting the lung parenchyma. In addition, one patient who relapsed with ILD after receiving durvalumab did not have solitary shadows located only outside the irradiation field at any time, indicating the possibility that local immune system changes in the lung due to radiation cannot be ruled out as the cause of relapse of ILD. Although various studies have been conducted on radiation therapy-induced changes in the immune system [19–21], much remains to be elucidated.

Moreover, in the cases reviewed in the present study, interstitial shadows extending outside the high-dose area were significantly associated with high HbA1c levels and with the severity of ≥ Grade 2 lung inflammation, whereas none of the lung dose–volume parameters showed a significant association. Although it has long been recognized that the finding of shadows extending outside the high-dose area on CT imaging indicates that RP might be severe, to the best of our knowledge an association with high HbA1c levels has not previously been reported. However, several studies have reported diabetes as a risk factor for RP [22, 23]; and it has also been reported that diabetic patients are in a chronic inflammatory state, with increased secretion of inflammatory cytokines such as TNF-α and IL-6 as well as increased production of reactive oxygen species by neutrophils [24–26]. Therefore, it can be inferred that the inflammatory response to radiation [15] is enhanced in diabetic patients, which may be associated with frequency and severity of RP. Normalization of blood glucose has been shown to normalize cytokine levels [27], and strict glycemic control before and after CRT may lead to risk reduction of RP.

Most of the present patients were treated with radiotherapy using 3D-CRT, but the number of patients treated with IMRT has been increasing in recent years. The greatest advantage of IMRT is the ability to reduce V_20_, but it should be noted that some increase of the lower-dose irradiated volume, e.g., V_5_ is inevitable, and it cannot be denied that this may lead to an increase in the potential risk of ILD/RP due to hypersensitivity to radiation. A secondary analysis of the large prospective trial RTOG 0617 showed that V_5_ was not involved in the development of Grade 3 or higher RP [28]. However, even before the approval of durvalumab, fatal RP of both lungs was reported in patients treated with IMRT [29], and a study of locally advanced lung cancer treated with CRT and durvalumab reported that V_5_ was the only factor significantly associated with pneumonitis free survival [30]. A retrospective Japanese study that evaluated CCRT using IMRT and durvalumab showed that V_5_ was significantly associated with ≥ Grade 2 pneumonitis. These results indicate that the interaction of durvalumab and extensive low dose irradiation to the lungs increases the risk of symptomatic pneumonitis [31]. The effect of radiation in the lung field, even at lower doses, should not be ignored and a detailed study of the patient’s pro-inflammatory factors and pattern of pneumonitis is warranted, especially in patients treated with immune checkpoint inhibitors after RT.

This study has several limitations. First, as the data were derived from one institution and a retrospective analysis was performed in a small sample, multivariate analysis of more than two factors was not performed and we cannot rule out the possibility that confounding factors were not sufficiently adjusted for. We used V_20_ as a covariate because it had been shown to be associated with severe radiation pneumonitis in many previous studies. We restricted the number of covariates to two for the multivariate analysis; however, given the limited number of events, there remains a risk of overfitting the model, which could compromise reliability of the estimated regression model. Second, 24% of patients could not receive chemotherapy for reasons other than respiratory disease. Third, patients who developed pneumonitis early after RT did not receive durvalumab, and these patients may have been undetected in the high-risk group for durvalumab.

## Conclusion

The results of the present study showed that durvalumab after CRT was effective and was not significantly associated with the incidence of ILD/RP itself. Although the incidence of ILD/RP that occurred in low-dose irradiated areas after RT was not high, it was shown to have an association with severe symptoms that interfered with administration of durvalumab, and had suggested risk factors of high HbA1c and/or glucose levels at the time of RT. Further studies on the clinical background, including diabetes at the time of RT, are warranted to improve the dose of durvalumab after CRT and ultimately the survival rate.

## Data Availability

The datasets used and/or analyzed during the current study are available from the corresponding author on reasonable request.

## Abbreviations

3D-CRT: three-dimensional conformal radiotherapy
AUC: area under the curve
CRP: C-reactive protein
CRT: chemoradiotherapy
CT: computed tomography
HbA1c: Hemoglobin A1c
ILD: interstitial lung disease
IMRT: intensity-modulated radiation therapy
KL-6: Krebs von den Lungen-6
LDH: lactate dehydrogenase
MLD: mean lung dose
NCI-CTCAE: National Cancer Institute Common Terminology Criteria for Adverse Events
NSCLC: non-small cell lung cancer
PD-L1: programmed cell death-ligand 1
PFS: progression-free survival
ROC: receiver-operating characteristic
RP: radiation pneumonitis
RT: radiotherapy
Vs5: the absolute lung volume spared from a 5 Gy dose
Vx: volume of lung parenchyma that receives x Gy or more
